# Relationship between Temperament and Stage of Lactation, Productivity and Milk Composition of Dairy Cows

**DOI:** 10.3390/ani11071840

**Published:** 2021-06-22

**Authors:** Ramūnas Antanaitis, Vida Juozaitienė, Vesta Jonike, Vytenis Čukauskas, Danguolė Urbšienė, Algirdas Urbšys, Walter Baumgartner, Algimantas Paulauskas

**Affiliations:** 1 Large Animal Clinic, Veterinary Academy, Lithuanian University of Health Sciences, Tilžės Str. 18, LT-47181 Kaunas, Lithuania; 2 Department of Biology, Faculty of Natural Sciences, Vytautas Magnus University, K. Donelaičio 58, LT-44248 Kaunas, Lithuania; vesta.jonike@vdu.lt (V.J.); danguole@gpk.lt (D.U.); Algirdas.Urbsys@lsmuni.lt (A.U.); algimantas.paulauskas@vdu.lt (A.P.); 3 State Enterprise Center for Agricultural Information and Rural Business, V. Kudirkos Str. 18-1, LT-03105 Vilnius, Lithuania; vytenis@vic.lt; 4 University Clinic for Ruminants, University of Veterinary Medicine, Veterinaerplatz 1, A-1210 Vienna, Austria; Walter.Baumgartner@vetmeduni.ac.at

**Keywords:** dairy cows, temperament, productivity, heath, heritability, phenotypic correlation, genetic correlation

## Abstract

**Simple Summary:**

Cattle temperament can be described as a response to changes in the environment and is crucial for successful herd management using innovative technologies. Despite the economic aspects of animal productivity and welfare, there is still a lack of objective evidence for a wider use of temperament in dairy cattle breeding programmes. The aim of this study was to evaluate the relationship between cow temperament and milk indices describing cow productivity, metabolic status and mastitis resistance. The coefficient of heritability of temperament was determined. Only a small part of the phenotypic changes in this indicator in the analysed population was associated with genetic factors; however, the correlation of cow temperament with milk lactose and somatic cells suggests that temperament could be used in sustainable breeding programmes, giving priority to animal welfare and health. A statistically significant decrease in temperament scores with increasing lactation periods was only found in primiparous cows. It is also argued that changes in milk production, milk composition and quality associated with mastitis and a cow’s metabolic status should be taken into account when assessing the cow’s temperament, as these factors can affect the welfare and behaviour of an animal, and therefore the expression and intensity of their reaction to their environment.

**Abstract:**

The aim of this study was to assess the relationship between temperament and milk performance in cows at different stages of lactation, describing their productivity, metabolic status and resistance to mastitis. This study showed that with increasing lactation, cows’ temperament indicators decreased (*p* < 0.001) and they became calmer. The highest temperament score on a five-point scale was found in cows between 45 and 100 days of lactation. In the group of pregnant cows, we found more cows (*p* = 0.005) with a temperament score of 1–2 compared with non-pregnant cows A normal temperament was usually detected in cows with lactose levels in milk of 4.60% or more and when the somatic cell count (SCC) values in cow milk were <100,000/mL and 100,000–200,000/mL, with a milk fat-to-protein ratio of 1.2. A larger number of more sensitive and highly aggressive cows was detected at a low milk urea level. In contrast to a positive phenotypic correlation (*p* < 0.05), this study showed a negative genetic correlation between the temperament of cows and milk yield (*p* < 0.001). Positive genetic correlations between temperament scores and milk somatic cells (*p* < 0.001) and milk fat-to-protein ratio (*p* < 0.05) were found to indicate a lower genetic predisposition in cows with a calmer temperament to subclinical mastitis and ketosis. On the other hand, the heritability of temperament (h^2^ = 0.044–0.100) showed that only a small part of the phenotypic changes in this indicator is associated with genetic factors.

## 1. Introduction

The European Union (EU) was the first region in the world to recognise the importance of animal welfare. Its animal welfare rules for dairy cows stem from Council Directive 98/58/EC from 20 July 1998, concerning the protection of animals kept for farming purposes, which provides general requirements for animal welfare in all farmed species based on the European Convention for the Protection of Animals kept for farming purposes drawn up within the Council of Europe [1].

For dairy cows, many measures of biological health can be used as indicators of animal welfare, e.g., those focused on disease, injury and reproductive problems [2]. According to a study conducted by Haskell et al. [3], animal temperament can be defined as a response to environmental or social stimuli, and there are a number of temperament traits in cattle that contribute to their welfare. Temperament can be defined based on the animal’s reactivity to human handling and response to novel objects or stressful situations. Assessments of cattle temperament can provide important information on the physical, physiological and psychological state of the animal, including immunity, stress level and metabolic processes [4].

The technique for assessing the temperament of cattle is based on the results of observations that allow animals to be described according to different types of nervous activity. The combinations of the generalised assessments obtained provide good justification for the temperament of the animals being tested, classifying them according to a generally accepted scheme (animals with strong balanced mobility, strong balanced inertia, severe imbalances and weak types of superior nervous activity are identified) [2].

Cattle temperament is important not just for animal welfare, but also for their productivity, health, longevity and farm profitability [5,6,7,8,9,10,11] therefore, it may make sense to use this indicator in dairy cattle breeding programmes. Yu et al. [12] argued that heritability coefficient values of cow temperament ranging from 0.17 to 0.40 may be sufficient for cattle selection. This statement has been confirmed by the research results of other authors. For example, the heritability coefficients of the Simmental breed by temperament range from 0.28 to 0.55 [13,14].

Farmers and scientists around the world are increasingly interested in genetic selection to improve behavioural performance indicators for cattle, particularly in terms of simplicity of management, wellbeing and adaptation in intensive production systems [4]. The temperament observed during milking is associated with cow health [14,15], longevity [16], milk productivity [17] and adaptation to milking systems [18,19].

Cattle temperament can be described based on their reactivity to human handling, novel objects or stressful situations, which is crucial for successful herd management with new innovative technologies; however, despite the economic and animal welfare aspects, as Chang et al. point out in their scientific publication [4], there is still a lack of objective indicators and limited inclusion of temperament in dairy cattle breeding programmes.

In this context, the aim of this study was to evaluate the relationship between temperament and the milk indicators of cows at different stages of lactation, describing their productivity, metabolic status and resistance to mastitis, thus expanding available knowledge on the usefulness of this indicator for sustainable breeding programmes and making animal welfare and health a priority.

## 2. Materials and Methods

### 2.1. Location, Animals

The experiment was carried out on four dairy farms of Holstein cows during the period from September 2019 to October 2020. A total of 2472 clinically healthy dairy cows (on average 2.67 ± 0.281 days in lactation and 203.04 ± 2.492 days in milk) were selected for an evaluation of their temperament. The average milk yield (MY) of cows was 34.47 ± 0.2811 kg, with a milk fat (MF) percentage of 4.39 ± 0.021%, protein (MP) of 3.61 ± 0.010%, milk fat-to-protein ratio (F/P) of 1.22 ± 0.005, milk lactose (ML) of 4.40 ± 0.005%, milk urea (MU) of 24.023 ± 0.145 mg/dL and milk somatic cell count (SCC) of 432.65 ± 18.660 thousand/mL.

The cows were kept in a free housing system and were fed a total mixed ration (TMR) throughout the year, two times per day at a set time, balanced according to the physiological requirements of a 550 kg Holstein cow providing 35 kg milk per day. TMR was formulated accordingly to meet or exceed the requirements. The ration was composed of a dry matter (DM) (%) value of 50.00, acid detergent fibre (% of DM) value of 19.00, neutral detergent fibre (% of DM) value of 28.00, non-fibre carbohydrates (% of DM) value of 39.00, crude protein (% of DM) value of 16.00 and net energy for lactation value of 1.7 (Mcal/kg). The average assessment of the body condition of cows on a five-point scale was 3.8 ± 0.16. The cows were milked by a DeLaval milking robot (DeLaval Inc., Tumba, Sweden).

### 2.2. Measurements

This study was undertaken in accordance with the provisions of the Law on Animal Welfare and Protection of the Republic of Lithuania. The study approval number is PK016965.

The temperament of each cow was assessed twice, during morning and evening milking, and the final score was determined on a five-point scale. The scores on the scale corresponded to the following characteristics of temperament: (1) very slow–very calm, (2) slow–calm, (3) normal, (4) sensitive–aggressive and (5) very sensitive–very aggressive [20].

Samples of cow milk (a sample of 45 mL from each cow) were taken after the second temperament assessment and transported from the farm to the laboratory for testing.

Testing of milk samples was performed at the “Pieno Tyrimai” (Kaunas, Lithuania) state enterprise, which to ensure the accuracy of milk parameter testing, has implemented a quality management system that complies with the requirements of the international standard ISO/IEC 17025:2005. The study of the contents of fat, protein and lactose in milk was carried out using a Lactoscope FTIR infrared meter (FT1.0. 2001; Delta Instruments, Drachten, The Netherlands), while the study of the total number of somatic cells in milk was performed using a Somascope MK2 heavy-duty counter (Delta Instruments, Drachten, The Netherlands), which was operated using the fluoro-opto-electronic method.

Metabolic disorders in the cows (subclinical ketosis, subclinical acidosis) were determined by a balanced milk fat-to-protein ratio and by milk lactose and milk urea levels. The assessment of the risk of mastitis in cows was carried out on the basis of a certain number of somatic cells being present in the milk.

### 2.3. Data Analysis and Statistics

Data were statistically evaluated using the R-4.0.3 package (Windows OS, Redmond, WA, USA). The normal distribution of all indicators was assessed using the Shapiro-Wilk normality test. Analysis of somatic milk cells was carried out with the logarithmic expression of this indicator [21] to achieve a normal distribution.

SCS = (log2 (SCC ÷ 100)) + 3(1)

The phenotypic data for cow temperament were statistically processed using the Wilcoxon–Mann–Whitney test and Spearman correlation.

According to lactation, cows were divided into three classes: first (*n* = 418), second (*n* = 971) and third or other lactations (*n* = 1083). The cows were grouped as follows by lactation period: 45–99 (*n* = 606), 100–200 (*n* = 581) and >200 days in milk (DIM) (*n* = 1285). According to reproductive status, cows were divided into two classes: non-pregnant (*n* = 522) and pregnant (*n* = 1950) cows. The temperament of the cows was evaluated by two experts (each expert evaluated 1236 cows). In evaluating cows from four farms (farm1, *n* = 624; farm 2, *n* = 334; farm 3, *n* = 926; farm 4, *n* = 588), two seasons (May–October, *n* = 1394 and November–April, *n* = 1078)) were included in the statistical model. We grouped the cows according to milk yield (MY) into two classes (MY < 30 kg, *n* = 1022 and MY ≥ 30 kg, *n* = 1450), according to milk fat/protein ratio (F/P) into three classes (F/P < 1.2, *n* = 990; F/P = 1.2, *n* = 462 and F/P > 1.2, *n* = 1020), according to milk lactose (ML) into six classes (ML < 4.00%, *n* = 112; ML = 4.00–4.19%, *n* = 208; ML = 4.20–4.39%, *n* = 626; ML = 4.40–4.60%, *n* = 921; ML = 4.60 = 4.80%, *n* = 505; ML = 4.80–5.00%, *n* = 100), according to milk urea (MU) into three classes (MU < 15 mg/dL, *n* = 257; MU = 15–30 mg/dL, *n* = 1674 and MU > 30 mg/dL, *n* = 541) and according to milk somatic cells (SCC) into five classes (SCC > 100,000/mL, *n* = 925; SCC = 100,000–200,000/mL, *n* = 506; SCC = 200,000–400,000/mL, *n* = 416; SCC = 400,000–600,000/mL, *n* = 241; SCC > 600,000/mL, *n* = 384). The Pearson chi-square test (χ^2^) of independence was used to assess the relationships between the assessments of cow temperament and the classes of these indicators.

For the study of heritability (h^2^) and genetic correlation (r_g_), PEST 4.2 (Multivariate Prediction and Estimation, 12 March 1999, Linux 2.0.36. Groeneveld E., Kovac M., Wang T. Department of Animal Sciences, University of Illinois) and VCE 4.2.5 (8 December 1998, Linux 2.0.34 i586, written by E. Groeneveld) programmes were used.

In the first model, we evaluated cow temperament (Y1), milk yield (Y2), milk fat (Y3), milk protein (Y4), milk fat-to-protein ratio (Y5), milk lactose (Y6), milk urea (Y7) and SCS (Y8) indicators. We calculated the genetic correlations and the heritability of these traits.

Y_ijklmn_ = L_i_ +D_j_ + R_k_ + E_l_ + HS_m_ + a_n_ + e_ijklmn_ (2)

The following effects and their statistical interpretations were applied in the first model, where L is the lactation number (fixed), D is the lactation period (fixed), R is the reproduction status of cows (fixed), E is there expert (fixed), HS is the herd season (fixed), a is the animal (additive genetic effect, random) and e is the error (random).

In the second model, we evaluated only the temperament of the cows:

Y_ijklmoprs_ = L_i_ +D_j_ + R_k_ + E_l_ + HS_m_ + M_n_+ F_o_ +T_p_+ S_r_ + a_s_ + e_ijklmnoprs_ (3)

The following effects were used in the second model, where L is the lactation number (fixed), D is the lactation period (fixed), R is the reproduction status of the cows (fixed), E is the expert (fixed), HS is the herd season (fixed), M is the milk yield class, F is the milk fat-to-protein ratio class (fixed), T is the milk lactose class (fixed), S is the milk SCC class (fixed), a is the animal (additive genetic effect, random) and e is the error (random).

All cows tested (*n* = 2472) were of known pedigree. We selected their ancestors (*n* = 34,608) of three generations from the national BLUP (Best linear unbiased prediction) database (State Enterprise Center for Agricultural Information and Rural Business) to assess the genetic parameters of animal temperament.

## 3. Results

In general, the herds did not show any kind of stress sign during the milking process and acted calmly and normally. The mean of the temperament scores for all cows was 2.53. Percentage distributions of individuals using the five-point temperament scores showed that 45.1% and 46.2% of cows were awarded scores of 2 and 3, respectively.

Based on correlation analysis, cow temperament scores tended to decrease with increasing lactation (r = −0.146, *p* < 0.001) and were negatively associated with the number of days in milk (r = −0.051, *p* = 0.011).

**Figure 1:**
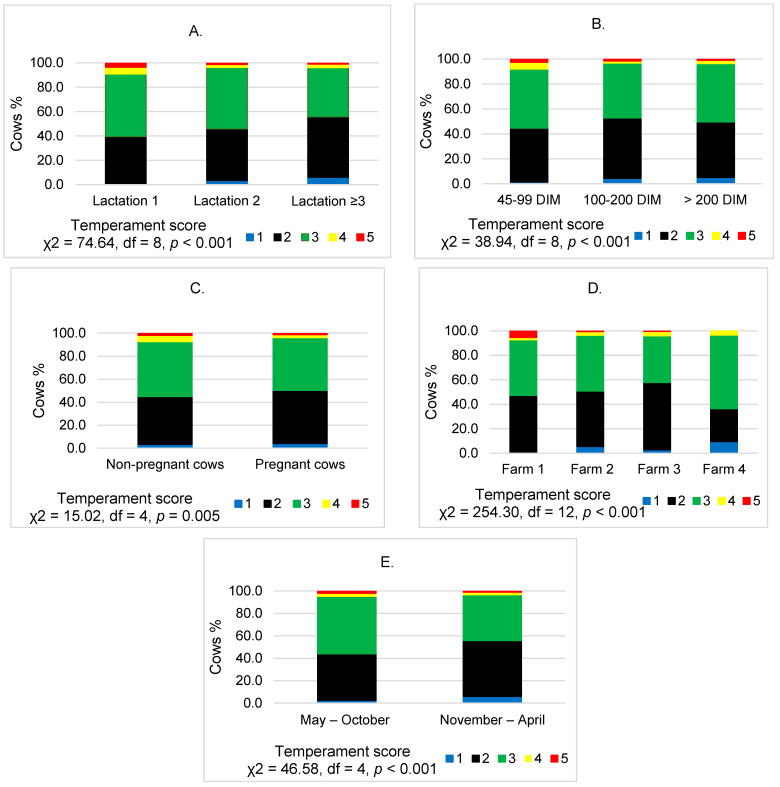
Cow temperament scores assessed by lactation, days in milk, reproductive status, farm and season. (**A**) Evaluation of cow temperament (scores) according to lactation; (**B**) Evaluation of cow temperament (scores) according to days in milk; (**C**) Evaluation of cow temperament (scores) according to reproductive status; (**D**) Evaluation of cow temperament (scores) according to farm; (**E**) Evaluation of cow temperament (scores) according to season. 1—very slow–very calm; 2—slow–calm; 3—normal; 4—sensitive–aggressive; 5—very sensitive–very aggressive.

The mean temperament score for the primiparous cows (2.73) was 0.17 points higher than that of the second lactation cows and 0.30 points higher than that of the multiparous cows. The evaluation of temperament scores by applying the Wilcoxon–Mann–Whitney test revealed significant differences between lactations (*p* < 0.001). The data analysis (Figure 1A) showed that with increasing lactation, the temperament of cows became calmer and the number of cows that received a score of 1–2 increased (1.2–2.7 times, *p* < 0.001).

The highest mean for the temperament score (2.65) was found in cows at 45 to 99 lactation days. During this period, the number of cows with a score of “1” was 3.02–3.46 times less than in later periods of lactation, while the number of cows with a score of “5” was 1.53–1.58 times more (*p* < 0.001). The data are summarised in Figure 1B. A statistically significant decrease in temperament scores with increasing DIM period was only observed in primiparous cows (*p* < 0.001).

In the group of pregnant cows, we found 1.11–1.23 times more cows (*p* = 0.005) with a temperament score of 1–2 compared with non-pregnant cows (Figure 1C). The Wilcoxon–Mann–Whitney test showed that the temperament score in non-pregnant cows had a higher evaluation value than in pregnant cows (*p* = 0.007).

The analysis showed that the assessment of the temperament of the cows depended on their farm (*p* < 0.001). The largest number of cows (7.3%) with a temperament score of 4–5 was on the first farm, while the largest number of cows with a score of 1–2 points (57.9%) was on the third farm (Figure 1D).

More (10.5%) cows with normal temperament were found in May and October (Figure 1E); however, during this period, the number of cows with 1–2 temperament points was 1.3 times more than in the period from November to April (*p* < 0.001).

**Figure 2:**
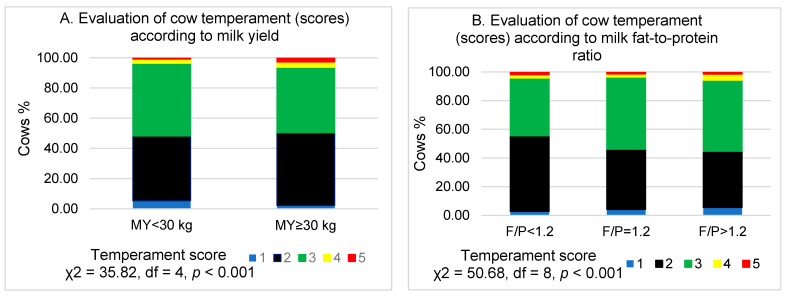

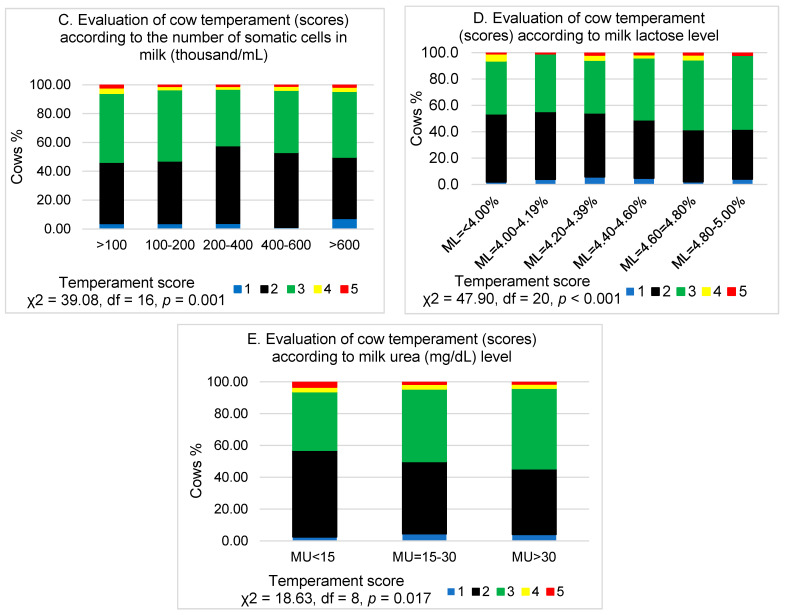
Evaluation of cow temperament scores according to milk indicators. F/P—milk fat-to-protein ratio; ML—milk lactose (%); MU—milk urea (mg/dL); SCC—milk somatic cell count (thousand/mL). (**A**) Evaluation of cow temperament (scores) according to milk yield; (**B**) Evaluation of cow temperament (scores) according to milk fat-to-protein ratio; (**C**) Evaluation of cow temperament (scores) according to the number of somatic cells in milk (thousand/ml); (**D**) Evaluation of cow temperament (scores) according to milk lactose level; (**E**) Evaluation of cow temperament (scores) according to milk urea (mg/dL) level. 1—very slow–very calm; 2—slow–calm; 3—normal; 4—sensitive–aggressive; 5—very sensitive–very aggressive.

As shown in Figure 2A, in the group of more productive cows, 3.13% fewer cows were identified to have a very slow–very calm temperament and 1.91% fewer cows had a very sensitive–very aggressive temperament (*p* < 0.001).

The mean value of the temperament score in cows with a milk fat-to-protein ratio of 1.2 was higher compared to cows with a milk fat-to-protein ratio of <1.2 (*p* = 0.012) and slightly lower than in cows with a milk fat-to-protein ratio > 1.2 (*p* = 0.528). Most cows (49.61–50.35%) with normal temperament were found at F/P = 1.2 and F/P > 1.2 levels. The greatest number of cows (5.69%) with a temperament evaluation of 4–5 was observed when the F/P > 1.2, while the greatest number of animals (55.45%) who received 1–2 points were found at F/P < 1.2 (Figure 2B).

The study showed that the temperament score in cows (*n* = 1012) with milk SCC ≥ 200,000/mL was lower (*p* < 0.001) than in cows (*n* = 1425) without signs of subclinical mastitis and with milk SCC < 200,000/mL. By grouping the cows into five classes according to the milk SCC (Figure 2C), the largest number of cows with a temperament score of 3 was found in groups with milk SCC < 100,000/mL (47.89%) and SCC = 100,000–200,000/mL (49.38%). The largest number of cows with very slow–very calm temperament was in the group with SCC > 600,000/mL (7.21%). Most animals with a very sensitive–very aggressive temperament were found in groups of cows with SCC < 100,000/mL (2.16%) or SCC > 600,000/mL (1.68%) in milk.

Cows with a normal temperament were most often seen when their milk lactose levels were 4.60–4.80% (52.87% of cows) and 4.80–5.00% (56.00% of cows). In other classes of lactose, the numbers of cows with a temperament score of 3 ranged from 39.94% to 47.01% (Figure 2D).

The class MU < 15 mg/dL had the smallest number of cows with a normal temperament (36.71%) and the largest number of very sensitive–very aggressive cows (3.38%). The analysis showed that the class with MU > 30 mg/dL had the largest number of cows with a temperament score of 3. When MU = 15–30 mg/dL in cow milk, this group had the most (4.42%) very slow–very calm cows compared with other groups according to MU (Figure 2E).

As can be seen from the data in Table 1, negative genetic correlations were found between the temperament of cows and their productivity and milk lactose (*p* < 0.01). In addition, positive genetic correlations between cow temperament and SCS, milk fat and protein percentage, and milk fat and protein ratio were calculated (*p* < 0.01). In contrast to genetic correlations, the phenotypic associations of temperament with cow milk and lactose were positive (*p* < 0.05). Positive phenotypic correlations were also found between cow temperament and milk urea and milk fat (*p* < 0.05).

Milk yield of cows was phenotypically and genetically negatively correlated with milk fat percentage and protein percentage (*p* < 0.01), while it was positively correlated with milk urea (*p* < 0.01). A negative phenotypic correlation and positive genetic correlation was found between milk yield and SCS (*p* < 0.01). Milk lactose positively correlated with the milk yield of cows (*p* < 0.01); however, the genetic relationship between these traits was stronger (1.31 times) compared to the genetic correlation. Milk lactose and SCS similarly correlated both genetically and phenotypically (r_g_ = −0.466 and r_p_ = −0.450, *p* < 0.01), but the negative genetic association between these traits was slightly stronger than the phenotypic association. Phenotypic (r_p_ = 0.336) and genetic correlations (r_g_ = 0.535) for the percentages of milk fat and protein were positive (*p* < 0.01).

The data in Table 1 showed that the heritability of the cow temperament (h^2^) was from 2.34 (milk urea, mg/dL) to 9.80 (milk lactose %) times lower than the heritability of other studied traits.

In the second model, in which we evaluated the heritability of cow temperament with additional factors, we found that its value was 2.21 times higher (h^2^ = 0.100) than in the first model.

## 4. Discussion

In the last few decades, greater attention has been focused on improving animal welfare in the European Union and globally. A practical approach can improve the temperament of farm animals, e.g., through the selection of breeding stock for good temperament [1].

Holstein, Jersey and Ayrshire cows with unfavourable temperament were found to have shorter longevity than cows with a calm temperament. Therefore, the scientific literature indicates that cow temperament is being studied to improve animal welfare and farm profitability [15]. Temperament is used to assess the breeding value of cattle [4]. The factors influencing the temperament of cattle are the breed, age, environment, habit and appraiser [4,22]. According to Hungarian data on Holstein–Friesian cows, Tőzsér et al. [23] found that average temperament scores revealed multiparous cows to have a better temperament than primiparous cows. The present study showed that the temperament of older lactating cows was calmer, indicating that they are better adapted to environmental and herd management conditions.

The reproduction state of cows can also be associated with the expression of temperament. Cows with excitable temperament had reduced reproductive performance [24]. In the group of pregnant cows; we found more cows with a temperament score of 1–2 compared with non-pregnant cows (*p* = 0.005).

A statistically significant (*p* < 0.001) decrease in temperament scores with increasing DIM was only observed in primiparous cows, which indicates that in later lactation periods they adapt better to the environment and become calmer. No such trends were observed in older cows. This confirms the claim by other authors [2,4] that habituation to milking and environmental conditions is important for an animal’s behaviour and responses to stimuli.

The assessment of bovine temperament may provide important information about an animal’s physical, physiological and psychological state, including immunity, stress levels and metabolic processes [4]. The metabolic status of cows can be described by the milk fat-to-protein ratio and milk lactose and urea levels [25,26].

In the present study, cows with a normal temperament were mostly found when their milk lactose level was 4.60% and above. A negative genetic correlation was calculated between cow temperament and the percentage of lactose in milk (r_g_ = −0.051, *p* < 0.001). According to the literature, the synthesis and concentration of lactose in milk are mainly influenced by the health of the cow’s udder; therefore, lactose negatively correlates with the number of somatic cells, which increases with inflammation of the udder (mastitis) [25,26]. The somatic cell count, an indicator of subclinical mastitis, is widely used in the EU by industry leaders and farmers to monitor milk quality and for official controls as an indicator of milk hygiene. The EU Directorate general health and safety review report states that this indicator is useful not only for milk hygiene, but also as a general indicator of animal welfare [1]. Orban et al. [27] and Fulwider et al. [28] reported that the number of somatic cells in the milk for quieter and more obedient cows was lower than that of more nervous cows. For nervous cows, the milk release process is slower [29]. The present study showed that the largest number of cows with a normal temperament (score 3) was found when the cow milk SCCs were in the ranges of <100,000/mL (47.89%) and 100,000–200,000/mL (49.38%).

The fat-to-milk ratio is a valuable indicator of lipomobilisation and negative energy balance in cows, as well as a good indicator of metabolic disorders [30,31]. The lowest and highest mean temperament scores were found in cows with milk fat-to-protein ratios showing subclinical acidosis (F/P < 1.2) or subclinical ketosis (F/P > 1.2), respectively. Most cows with a normal temperament were in the group with a milk fat-to-protein ratio of 1.2. Positive genetic correlations between temperament scores and the milk fat-to-protein ratio and somatic cell count in milk (r_g_ = 0.013 and r_g_ = 0.201, respectively, *p* < 0.01) were found to indicate a lower genetic predisposition of cows of a calmer temperament to ketosis and subclinical mastitis.

The optimum concentration of the final nitrogen metabolite, called urea, in cow milk should be in the range of 15–30 mg/dL [32,33]. A larger number of more sensitive and highly aggressive cows were detected to have a low milk urea level compared with other groups. This suggests that an animal’s wellbeing and state of health affect its sensitivity and response.

A previous study confirmed a favourable genetic correlation (−0.40) between cow milk yield (adjusted for milk fat content) and temperament [34]. In contrast to the positive phenotypic correlation (r_p_ = 0.043, *p* < 0.05), the present study showed a negative genetic correlation between the temperament of cows and their productivity (r_g_ = −0.113, *p* < 0.001).

Chang et al. [4] reported that according to studies published between 1960 and 2019, the temperament of dairy cattle is a moderately heritable trait, with a wide variation in heritability estimates depending on the indicator trait (0.002–0.47), while phenotypes categorised with a smaller number of indicators have lower heritability. Recent studies have shown that the use of automatic milking systems (AMS) is facing new challenges in cow herds. Wethal and Heringstad [19] have published promising genetic parameters of new properties that describe the effectiveness of milking and milking temperament when using AMS. Due to the favourable genetic parameters identified, many of the temperament characteristics of cows evaluated during milking are useful for the genetic evaluation and improvement of animals.

The heritability of temperament results (h^2^ = 0.044–0.100), as calculated in this study, showed that only a small number of the phenotypic changes in this indicator in the analysed population are associated with genetic factors. Favorable genetic correlations of cow temperament with milk lactose and somatic cells suggest that genetic temperament enhancement may help improve cow health.

## 5. Conclusions

A statistically significant (*p* < 0.001) decrease in temperament scores was found with increasing lactation periods in primiparous cows. Normal temperament was usually observed in cows with lactose levels in milk of 4.60% or more and when the SCC in milk was < 200,000/mL. The lowest and highest temperament scores were found in cows when their milk fat-to-protein ratio was unbalanced and showed subclinical acidosis or subclinical ketosis. A positive genetic correlation was detected between temperament scores and milk somatic cells, while the ratio of milk fat-to-protein indicated a lower genetic predisposition of calmer temperament cows to subclinical mastitis and ketosis.

This study also suggests that a cow’s reproductive status and changes in milk productivity, composition and quality related to subclinical mastitis and metabolic status should be taken into account when assessing cow temperament, as these may affect animal welfare and behaviour, and thus the expression and intensity of their response to the environment.

## Figures and Tables

**Table 1:** Phenotypic (below the diagonal) and genetic (above the diagonal) correlations of cow temperament with milk indices and their heritability (diagonally).

Indices	Temperament	MY (kg)	MF (%)	MP (%)	F/P	ML (%)	MU (mg%)	SCS
Temperament	0.044	−0.113 **	0.030 **	0.011 **	0.013 **	−0.051 **	0.042 **	0.201 **
MY (kg)	0.043 *	0.231	−0.293 **	−0.399 **	0.009 **	0.401 **	0.205 **	0.174 **
MF (%)	0.040 *	−0.488 **	0.310	0.336 **	0.498 **	−0.095 **	0.018 **	0.142 **
MP (%)	−0.024	−0.485 **	0.535 **	0.342	−0.062 **	−0.045 **	0.018 **	0.280 **
F/P	0.073 **	−0.250 **	0.815 **	−0.039	0.113 **	0.004 **	0.052 **	0.023 **
ML (%)	0.038 *	0.305 **	−0.109 **	−0.264 **	0.062 *	0.431	0.063 **	−0.466 **
MU (mg/dL)	0.045 *	0.157 **	0.011	−0.047 *	0.041 *	0.080 **	0.103	−0.071 **
SCS	−0.047 *	−0.311 **	0.108 **	0.212 **	−0.020	−0.450 **	−0.120 **	0.192

MY—milk yield (kg); MF—milk fat (%); MP—protein (%); F/P—milk fat-to-protein ratio; ML—milk lactose (%), milk; MU—milk urea (mg/dL); SCS = (log2 (SCC ÷ 100)) + 3; SCC—milk somatic cell count (thousand/mL). Correlation coefficients are statistically reliable: * *p* < 0.05, ** *p* < 0.01.

## Data Availability

The data presented in this study are available within the article.
